# High- and low-Molecular Weight oat Beta-Glucan Reveals Antitumor Activity in Human Epithelial Lung Cancer

**DOI:** 10.1007/s12253-017-0278-3

**Published:** 2017-07-29

**Authors:** Anna Choromanska, Julita Kulbacka, Joanna Harasym, Remigiusz Oledzki, Anna Szewczyk, Jolanta Saczko

**Affiliations:** 1 Department of Medical Biochemistry, Wroclaw Medical University, Chalubinskiego 10, 50-368 Wroclaw, Poland; 2 Department of Food Biotechnology, Wroclaw University of Economics, Komandorska 118-120, 53-345 Wroclaw, Poland; 3 Department of General Zoology, Zoological Institute, University of Wroclaw, Sienkiewicza 21, 50-335 Wroclaw, Poland

**Keywords:** Oat beta-glucan, Viability, Lung cancer, Keratinocytes, Oxidative stress, Cytoskeleton

## Abstract

Beta-glucans are widely used in treatment, cosmetics, and the food industry. Glucans play a significant role in activation of the immune and antioxidant system and inhibiting tumor proliferation. In the current study the antitumor activities of new high and low molecular weight beta-glucan derived from oats were investigated in two human lung cancer cell line (A549, H69AR) and normal keratinocytes (HaCaT). The effect of high and low molecular weight beta-glucan from oat was evaluated by cellular viability assessment, lipid peroxidation and manganese superoxide dismutase evaluation and cytoskeleton visualisation. Additionally the level of red blood cells hemolysis was performed. Our results indicate strong anti-tumor properties of new beta-glucan from oat and at the same time no toxicity for normal cells.

**Electronic supplementary material:**

The online version of this article (doi:10.1007/s12253-017-0278-3) contains supplementary material, which is available to authorized users.

## Introduction

Despite of the advances methods in medicine and the dynamics of biochemical and biotechnological techniques, it is more often reached up to the sources of natural medicine. Herbal medicine, principles of rational nutrition concerns of growing as an alternative way of treatment and support for pharmacological therapy. In recent years interest in the use of plants for the production of compounds of pharmacological is increased. Additionally various studies have shown that components of plant can prevent disease, especially cancer. Using analytical methods for pharmacodynamic and which are used to assess the therapeutic properties and their actions, we get more and more data on the therapeutic efficiency of herbal supplements [1–3]. Recently, one of the active ingredients responsible for the anticancer therapy of many of these herbs was found to be a form of complex polysaccharides known as “beta-D-glucan”. It is non cellulosic polymers, elevating of the cellular walls, of beta-glucose, which is glycoside in position beta (1–3), (1–4) or beta (1–6). It occurs in the form of long-chain, three-dimensional molecule polysaccharide side chains of glucose molecules built [4]. Beta-glucans are isolated mainly from different fungi. However there are obtained from other sources, such as cereals, barley, bacteria or seaweeds. Beta-glucans, which are isolated from different sources characterized similar biological properties but different structures and solubility in water or other solvents [5]. The variety of materials from which beta-glucan is obtained, makes it possible to get a large number formulations of similar or different parameters. Immunomodulatory and anti-cancer properties of beta-glucans result from their structure, molecular weight, degree of branching, conformation and its behavior in gastrointestinal tract [6]. Moreover, these properties depend on an isolation procedure of beta-glucan. Beta-glucans belong to well-known biologic modifiers, which play immunostimulatory function against infections and cancer [7, 8]. This compound activated the response of adaptive immune cells such as CD4+ or/and CD8+ T cells and in addition B cells. It is one of the possible mechanisms of protective and anticancer action. However, the mechanisms of anticancer activities of beta-glucan seem to be multicomplex and still understood. Beta-glucan extracted from cell wall of fungi is characterized by low purity and additionally causes unfavorable effect. Moreover, there are water insoluble fraction [9].

Recently more remark is concentrated on beta-glucan from cereals mainly from oat. This soluble fibre fraction is the greatest find of recent years [10]. Numerous researches confirmed the significant anticancer activity of beta-glucan [11–13]. In the treatment of breast cancer, and also in areas undergoing irradiation after amputation, more rapid healing was obtained of inflammation. The use of beta-glucan in these appeared unaffected healthy tissue [14]. Still is well documented only the immune activation role of oat beta-glucan in killing cancer cells [15, 16]. We postulated that anticancer activity of oat beta-glucan is more complicated and complex. Many of the compounds used in the methods of standard anticancer therapy activated oxidative stress in cancer cells, which leads to cell death through lipid and thiol groups of protein peroxidation. Moreover, beta-glucan can influence on cells cytoskeleton and antioxidant preventing system.

The aim of the study was to evaluate the cytotoxicity and antitumor activities of high-molecular weight (HMW) and low-molecular weight of beta-glucan (LMW) derived from oats, based on the two cell lines, normal and cancerous. Additionally, the effect of the plant polysaccharides, such the beta*-*glucan, on human red blood cell hemolysis as an indicator of membrane stability, was investigated.

## Material and Methods

### Cell Culture

Three human cell lines, (purchased from the American Type Culture Collection Cell-ATCC-American Type Culture Collection) were used in this investigations: HaCaT - normal, immortal cell line of the transformed phenotype in vitro, the first stable line of adult human keratinocytes presenting normal differentiation. That is typical adherent cell line, growing in monolayer. A549 - human epithelial lung cancer cell line, derived from the 58-year-old Caucasian man. That is typical adherent cell line, growing in monolayer. H69AR - human multidrug resistant small cell lung cancer cell line, derived from the 55-year-old Caucasian man. H69AR cells begin to grow as aggregates which attach as domed patches. Most cells flatten into an epithelial monolayer, however some areas have piling of cells. Even when cells are healthy and well-attached, there are many viable floating cells.

All cell lines were grown in polystyrene flasks with 25 cm^2^ cell culture surface (Eppendorf, Germany) as a monolayer in Dulbecco modified Eagle medium (DMEM, Sigma-Aldrich, USA) for HaCaT and A549 cell line or RPMI-1640 Medium, (Gibco, USA) for H69AR cell line. Mediums were enhanced 10% (DMEM) or 20% (RPMI-1640) fetal bovine serum (FBS, Sigma-Aldrich, USA) and 50 μg/ml streptomycin (Sigma-Aldrich, USA). Cells were incubated at 37 °C in 5% CO_2_. Before the every experiments the cells were removed by 0.25% trypsin with 0.02% EDTA (Sigma-Aldrich, USA).

### HMW and LMW Beta-Glucans Recovery Procedure

In this examination beta-glucan from oats was derivedin form of white powder. HMW and LMW beta-glucan was obtained at the University of Economics in Wroclaw. HMW oat beta-glucan was obtained due to procedure described elsewhere [17] with beta-glucanase inactivation during lipid removal step, alkaline extraction, protein removal in isoelectric point, solution neutralization to pH = 7,0 and beta-glucan precipitation with ethanol. Low molecular oat 1–3, 1–4–D-beta-glucan was manufactured due to procedure described elsewhere [10] with multistep freeze-milling of raw materials (20% beta-glucan fiber, Microstructure, Poland), fat removal with ethanol extraction, alkaline extraction (pH 8–10) of beta-glucan and oat proteins, protein precipitation and separation in isoelectric point, and finally beta-glucan precipitation with ethanol in equilibrium. Beta-glucan preparations was then dried for 24 h. Purity was determined with according to AOAC 995.16 method with test kit (Megazyme, Ireland) and was 84%. Molecular weight of oat beta-glucan was measured with HPLC-SEC with guard column (OHpak SB-G, Shodex), a GPC column (SB-806 M HQ, Shodex) and was 69,650 g/mol. Firstly the stock solution of beta-glucan was prepared. Two mg of beta-glucan was dissolved in 1 ml of sterile distilled water and one drop of 10% NaOH was added. Then the stock solution was incubated at 37 °C for 24 h. The different concentrations of this compound were used to the studies (5 μg/ml, 10 μg/ml, 20 μg/ml, 25 μg/ml, 50 μg/ml, 75 μg/ml, 100 μg/ml, 150 μg/ml and 200 μg/ml)***.***

### Cellular Viability - MTT Assay

The viability of cells was determined by MTT assay (Sigma-Aldrich, USA) after experiments with different concentrations of beta-glucan. The MTT assay was used to estimation of mitochondrial metabolic function through the measurement of mitochondrial dehydrogenase. For the experiment the cells were seeded into 96-well micoculture plates at 1 × 10^4^ cells/well and grown overnight. After incubation with selected concentrations of LMW and HMW beta-glucan the experiments were realized according to the manufacture’s protocol. Those cells were incubated 24 h. The absorbance was determined using a multiwell scanning spectrophotometer at 570 nm (Enspire Perkin Elmer Multiplatereader, USA). Mitochondrial metabolic function was expressed as a percentage of viable treated cells in relation to untreated control cells.

### Lipid Peroxidation

The measurement of lipid peroxidation assay is based on the reaction of malondialdehyde with thiobarbituric acid (TBA). The cells were treated with selected concentrations of LMW and HMW beta-glucan. After 24 h incubation the cells were removed by trypsinization and suspended in phosphate buffered saline (PBS, Sigma Aldrich, USA). The final product of lipid peroxidation, malondialdehyde (MDA), reacts with TBA to form a colored complex. The level of MDA was measured by the absorbance at a wavelength of 535 nm. The concentration of MDA was quantified spectrophotometrically based on a set of MDA standards of known concentration [18].

### Immunofluorescencent Assessment of Cytoskeleton – CLSM Study

The confocal laser scanning microscopy (CLMS) was used to assess the morphology of treated cells. A549, H69AR and HaCaT cells were prepared for immunofluorescence reaction. The cells were grown on coverslips, than fixed 4% paraformaldehyde (PFA, Sigma-Aldrich, USA) in PBS, permeabilized with 0.5% Triton X-100 (Sigma-Aldrich, USA) in PBS (*v*/v) for 5 min. And blocked with 1% FBS in PBS (for 30 min. at room temperature). The cells were washed in PBS on the every steps of procedure. The following antibodies were used: primary antibody monoclonal anti-F-actin antibody produced in mouse (overnight incubation at 4 °C; 1:100; Sigma-Aldrich, USA); secondary antibody goat anti-mouse IgG FITC conjugated (for 60 min. at room temperature; 1:50; Sigma-Aldrich, USA). DNA was stained with DAPI (4,6-diamidino-2-phenylindole; 0.2 μg/ml in mounting medium with Mowiol-0.1% and DABCO). For imaging, Olympus FluoView FV1000 confocal laser scanning microscope (Olympus, Japan) was used. The images were recorded by employing a Plan-Apochromat 60× oil-immersion objective.

### Immunocytochemical MnSOD Evaluation

Immunocytochemistry was performed using the ABC method. The cultures were fixed and dehydrated using 4% PFA during 10 min. The samples were then permeabilized and blocked by incubation with 0.1% Triton X-100 in PBS. The enzymes expression were visualized with polyclonal antibody (1:100, anti-MnSOD; SOD 2; Santa Cruz, USA). For conventional bright-field microscopy (peroxidase-ABC labelling), the samples were incubated with a diaminobenzidine-H_2_O_2_ mixture to visualize the peroxidase label and counterstained with haematoxylin for 30 s. The samples were analyzed with the upright microscope (Olympus BX51, Japan). Stained cells were determined by counting 100 cells in randomly selected fields. The result was judged positive if staining was observed in more than 5% of cells. The intensity of immunohistochemical staining was evaluated as: (−) negative, (+) weak, (++) moderate and (+++) strong.

### Red Blood Cells Hemolysis

The level of hemolysis was determined by spectrophotometry. Red blood cell hemolysis was induced using sodium chloride solutions of various degrees of hypotonicity. The effect of each type of beta-glucan (LMW and HMW beta-glucan fraction) on erythrocytes hemolysis was examined at two concentrations (300 μg/ml and 400 μg/ml) and incubation periods of 24 h.

#### Preparation of red Blood Cells Suspensions

Blood samples from healthy volunteers were obtained in heparinized tubes. The red blood cells were separated from leukocytes by centrifugation (3000 rpm for 10 min.) and subsequently, washed three times with 0.9% sodium chloride solution (saline). Then prepared mixture of erythrocytes was filling by saline until the correct hematocrit for an individual subjects (approximately 40% in healthy females and 43% in healthy men) [19].

#### Preparation of Beta-Glucan Solutions

The solutions of beta-glucan (LMW and HMW beta-glucan fraction) were prepared by dissolving the requisite amount of solute in mixture of erythrocytes to obtain the desired concentrations (300 μg/ml and 400 μg/ml).

Each of the prepared test mixtures of red blood cells with beta-glucan (0.02 mL) was introduced into a tube with respective concentration of hypotonic sodium chloride solution. Then the tubes were incubated for 0.5 h at 23 °C (at room temperature) and then centrifuged (2000 rpm for 5 min.). The absorbance of the supernatant solution was measured spectrophotometrically at 540 nm using a microplate reader (EnSpire, PerkinElmer, USA) [20].

### Statistical Analysis

Data are reported as mean ± standard deviation (SD). Analysis utilized the one-way repeated measures analysis of variance (ANOVA). As a post-hoc test when results of the above test were significant Fisher’s Least Significant Difference (LSD), Tukey’s Honestly Significant Difference (HSD) and Scheffe’s test were applied, where Scheffe’s is the most conservative of the three. A level of *P* < 0.05 was considered to be statistically significant. The results from analysis are presented in supplementary material (SM).

## Results

### Cellular Viability

**Fig. 1:**
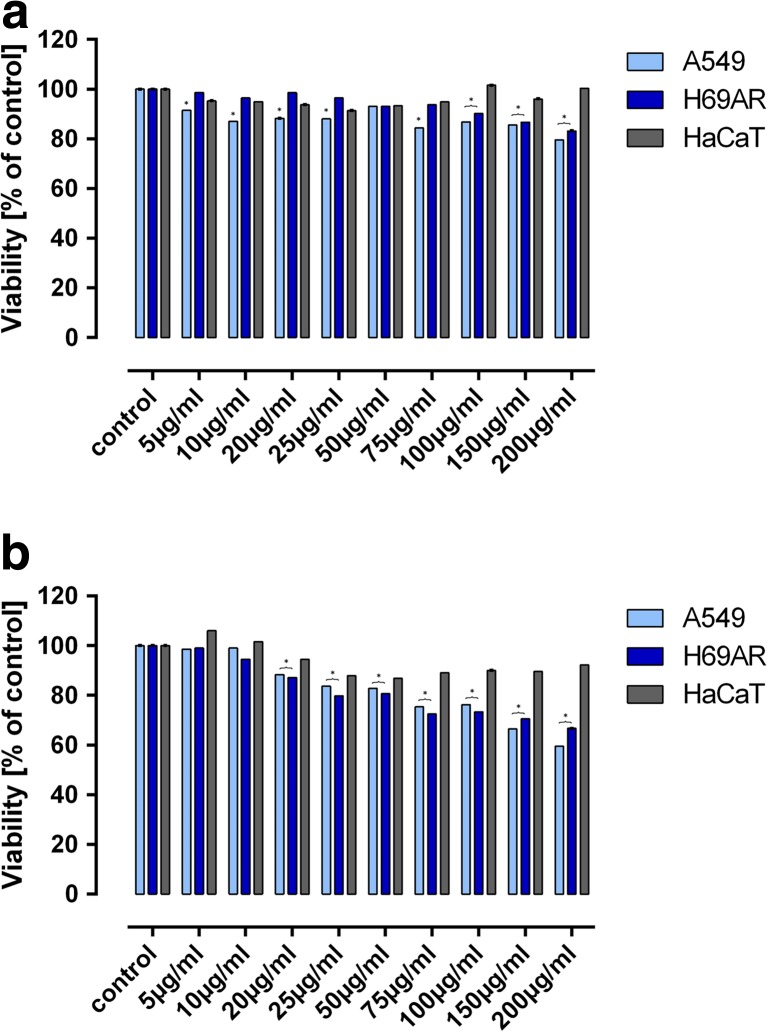
The effect of and LMW (panel A) and HMW (panel B) beta-glucan on the cells viability. Error bars shown are means ±SD for *n* ≥ 3.*P ≤ 0.05

The MTT assay showed that LMW beta glucan derived from oats hasn’t demonstrated cytotoxic effect against normal HaCaT cells, while in A549 and H69AR cells it caused slightly decreased in cells viability (to about 80%) (Fig. 1a). In turn HMW beta-glucan hasn’t demonstrated cytotoxic effect against normal cells (Fig. 1b), but for cancerous it cytotoxic. The cytotoxicity was increased with rising concentration of beta-glucan. The greatest decrease of cells viability was observed for 200 μg/ml and reached about 60% for A549 cells and 70% for H69AR. That cells were more sensitive to HMW beta-glucan from oats (Fig. 1b).

### Lipid Peroxidation

**Fig. 2:**
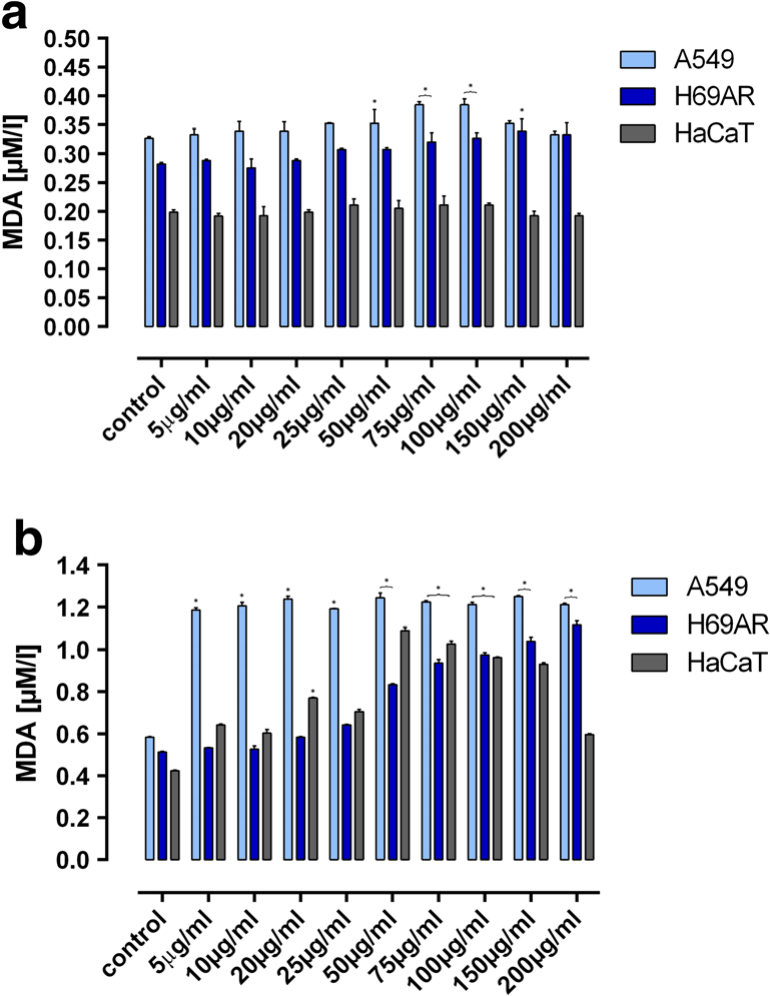
The effect of and LMW (panel A) and HMW (panel B) beta-glucan on the MDA level in cells. Error bars shown are means ±SD for *n* ≥ 3. *P ≤ 0.05

LMW beta glucan didn’t cause significant changes in MDA level in tested cells (Fig. 2a), where HMW beta glucan clearly caused increase of MDA level in all tested cell lines. In A549 cell line this increase was almost the same in all HMW beta-glucan concentrations. In H69AR cell line MDA level was increasing with increase of HMW beta-glucan concentrations, while for HaCaT cells MDA level was increasing also, but only to 50 μg/ml concentration of HMW beta-glucan. Above that concentration the level of MDA was decreasing (Fig. 2b).

### Assessment of Changes in the Cell Cytoskeleton

**Fig. 3:**
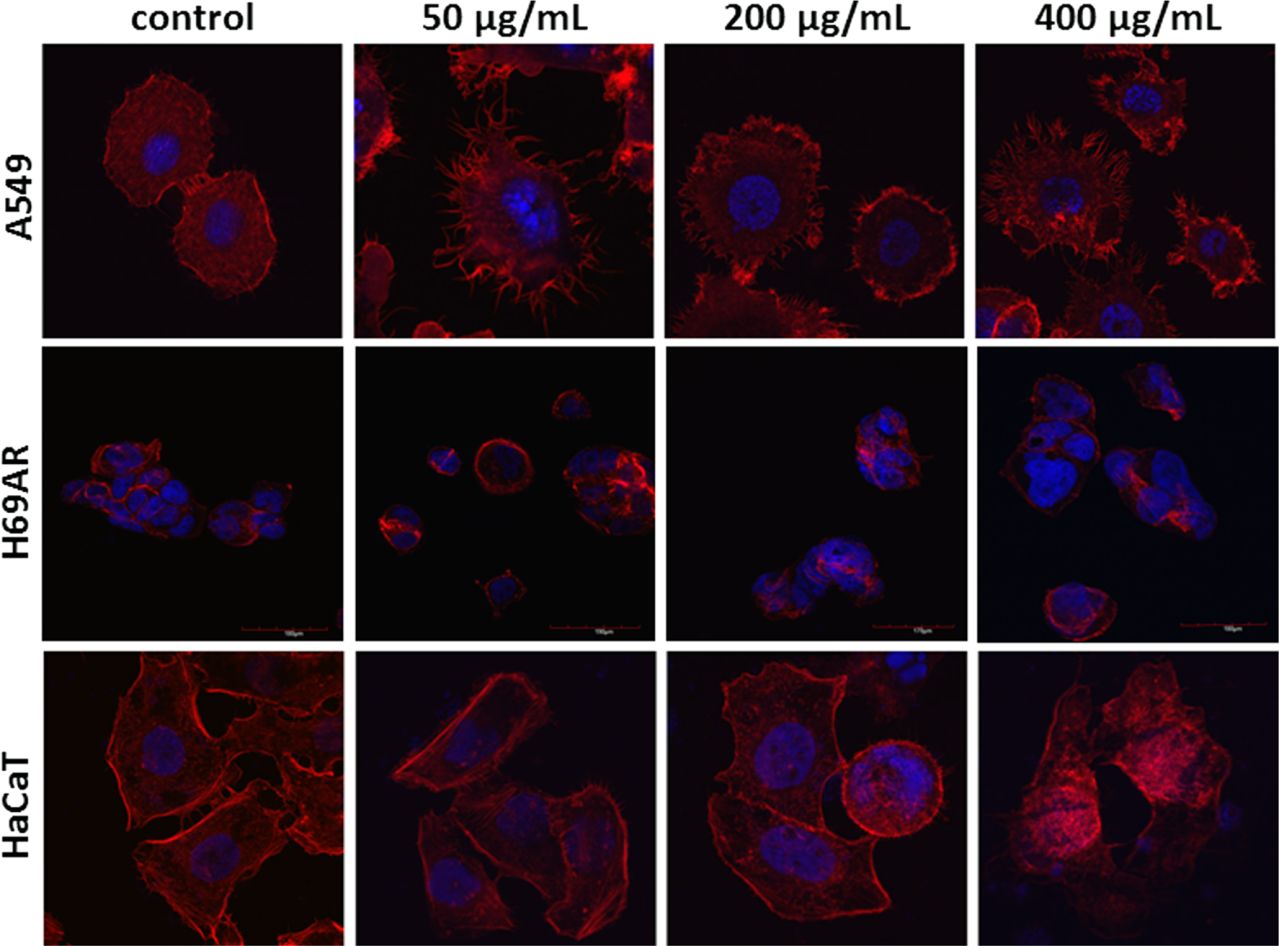
The cytoskeleton visualization in A549, H69AR and HaCaT cell line after treatment with LMW beta-glucan. Red fluorescence: F-actin fibers, blue fluorescence: cell nucleus

**Fig. 4:**
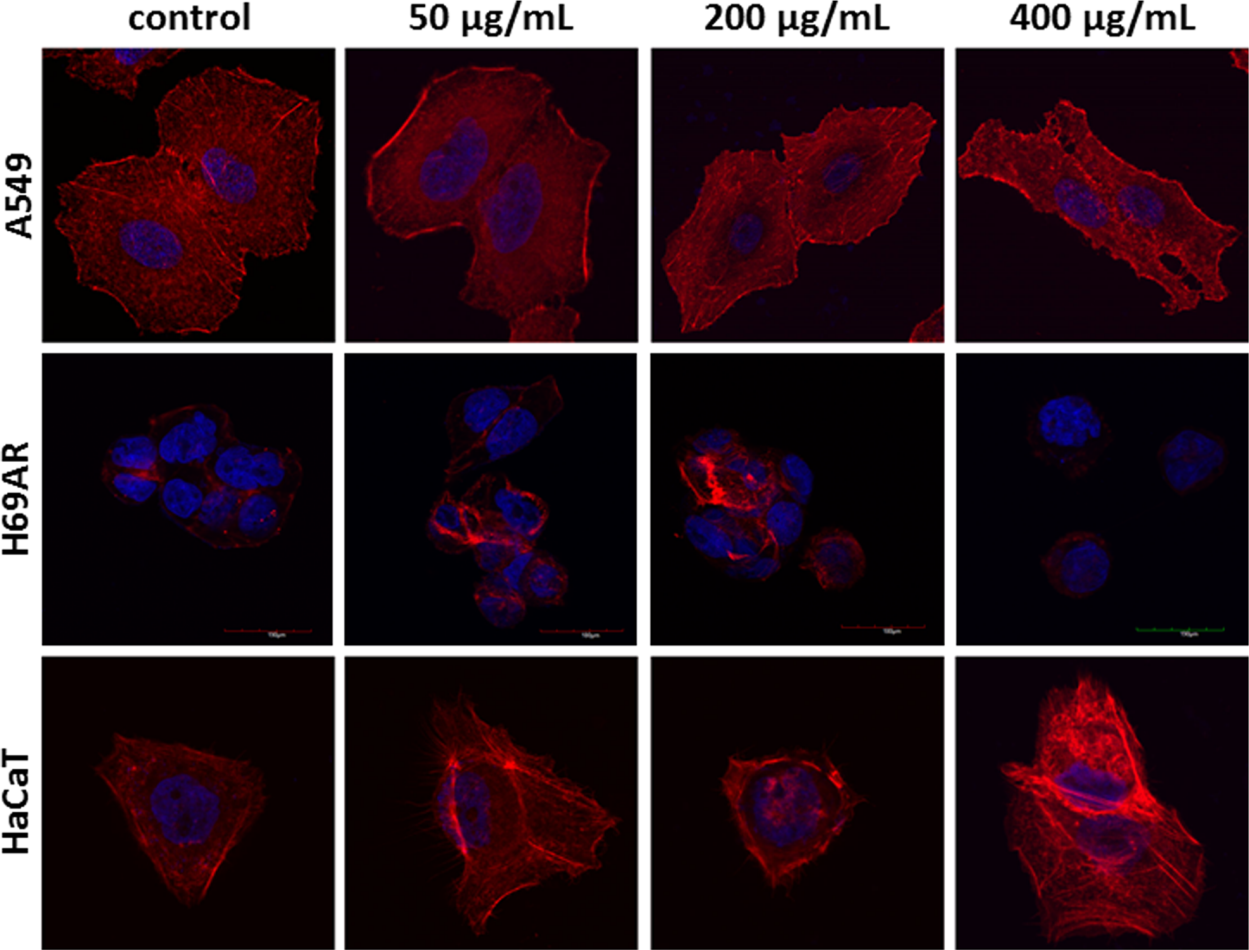
The cytoskeleton visualization in A549, H69AR and HaCaT cell line after treatment with HMW beta-glucan. F-actin fibers, blue fluorescence: cell nucleus

A low (50 μg/ml) as well as high (200 and 400 μg/ml) concentrations of LMW beta-glucan cause dramatic changes in cell morphology manifested by nucleus perturbations such as nuclear blebbing and abnormal chromatin condensation in A549 and HaCaT cell lines (Fig. 3). In H69AR cells LMW beta-glucan did not cause significant changes in the cytoskeleton (Fig. 3). In case of a HMW beta-glucan abnormalities in actin fibers arrangement were observed at each tested concentrations in H69AR and HaCaT cell line (Fig. 4).

### MnSOD Expression

**Fig. 5:**
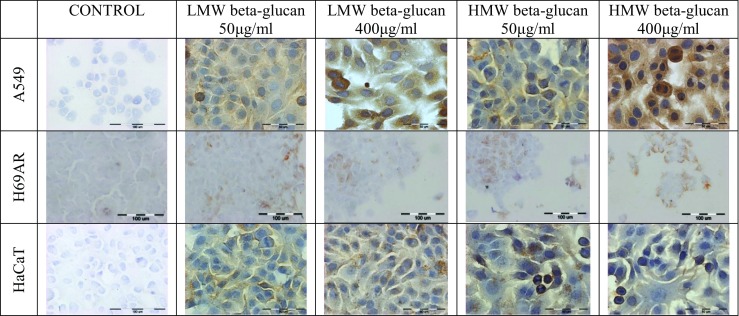
MnSOD expression in A549, H69AR and HaCaT cell line after treatment with LMW and HMW beta-glucan (×400)

LMW and HMW beta glucan increased the expression of MnSOD compared to control cells in all tested cell lines, but the highest expression was visible in A549 cells after incubation with LMW beta-glucan for concentration 50 μg/ml, 400 μg/ml (90 and 100% respectively) and HMW beta-glucan for 400 μg/ml (100%). HaCaT and H69AR cells demonstrated lower expression than A549 cells for experimental conditions (Fig. 5 ant Table 1).

**Table 1:** The immunocytochemistry analysis of MnSOD expression in normal HaCaT and lung cancer A549 and H69AR cell lines after exposure to the LMW and HMW beta-glucan

		The intensity of immunocytochemical staining	% of positively stained cells
A549	control	−	0
	LMW beta-glucan 50 μg/ml	+	90
	LMW beta-glucan 400 μg/ml	++	100
	HMW beta-glucan 50 μg/ml	++	40
	HMW beta-glucan 400 μg/ml	++/+++	100
H69AR	control	−	0
	LMW beta-glucan 50 μg/ml	+	30
	LMW beta-glucan 400 μg/ml	+	30
	HMW beta-glucan 50 μg/ml	+/++	50
	HMW beta-glucan 400 μg/ml	+/++	50
HaCaT	control	−	0
	LMW beta-glucan 50 μg/ml	+	40
	LMW beta-glucan 400 μg/ml	+	80
	HMW beta-glucan 50 μg/ml	+/++	50
	HMW beta-glucan 400 μg/ml	+	50

Results were presented as the percentage of stained cells. The evaluation of stained reaction: (−) negative, no reaction, (+) weak, (++) moderate and (+++) strong. Result present as a mean of cells number counted from 3 fields with ± SD (×400)

### Red Blood Cells Hemolysis

**Fig. 6:**
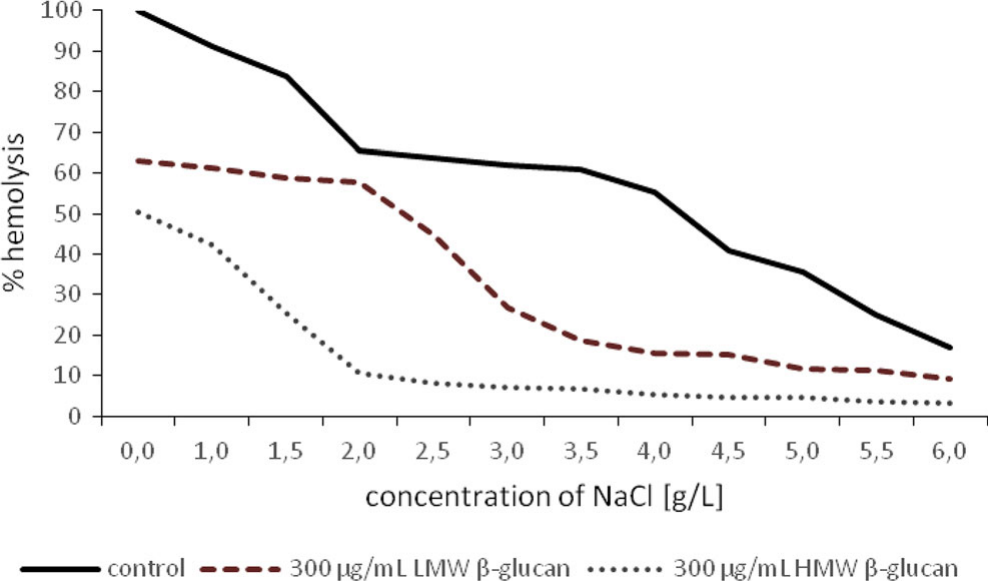
Inhibition of hypotonic sodium chloride solution-induced hemolysis of human erythrocytes by beta*-*glucan (300 μg/ml). On the graph marked course of hemolysis with LMW beta glucan (dash line) and HMW beta glucan (small dots line) after 24 h of incubation. Course of hemolysis without beta*-*glucan (control) marked on the graph solid line

**Fig. 7:**
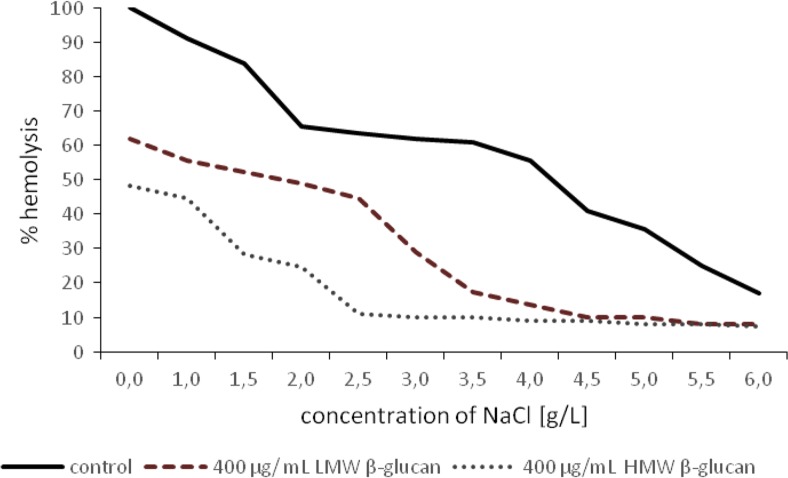
Inhibition of hypotonic sodium chloride solution-induced hemolysis of human erythrocytes by beta-glucan (400 μg/ml). On the graph marked course of hemolysis with LMW beta glucan (dash line) and HMW beta glucan (small dots line) after 24 h of incubation. Course of hemolysis without beta-glucan (control) marked on the graph solid line

Our results showed that the inhibition of hemolysis in the presence of beta*-*glucan and the inhibition of hemolysis was concentration dependent (Figs. 6 and 7). The highest level of inhibition of hemolysis was also observed at the higher of the two tested concentrations of HMW beta-glucan (Figs. 6 and 7). The red blood cells hemolysis in water free of sodium chloride (in distilled water) was inhibited by 36,95%, 49,5%, 38,18% and 51,64% at 300 μg/mL concentrations of LMW beta-glucan*,* 300 μg/ml concentrations of HMW beta-glucan, 400 μg/ml concentrations of LMW beta-glucan*,* and 400 μg/ml concentrations of HMW beta-glucan*,* respectively. Results were considered statistically significant at *p* > 0.05. The concentrations of chloride solution needed to cause hemolysis of red blood cell in the presence of beta*-*glucan are shown in Table 2.

**Table 2:** The percent hemolysis of human erythrocytes treated with exemplary hypotonic sodium chloride solution. The rate of hemolysis decreased with increasing concentrations of the protective agent – oat beta*-*glucan

Form and concentration of beta-glucan [μg/ml]	Concentration of hypotonic sodium chloride solution [g/L]	% hemolysis of human erythrocytes
LMW 300	0	63,05
HMW 300	0	50,50
LMW 400	0	61,82
HMW 400	0	48,36
LMW 300	1	61,45
HMW 300	1	42,36
LMW 400	1	55,64
HMW 400	1	44,55
LMW 300	3	26,73
HMW 300	3	7,26
LMW 400	3	29,09
HMW 400	3	10,00

## Discussion

Beta-glucan is frequently a natural products used in medicine, cosmetics, and the food industry. Glucans can activate the immune system and demonstrate antitumor effects. The mechanisms by which beta-glucan can destroy cancer cells are very complex and still not fully understand. Some results suggest that immunomodulatory and anti-cancer features of b-glucans consequence from their structure, molecular weight, degree of branching and conformation. At present most investigation has been focus on cereals’ extract due to its good water-solubility. It is commonly known that beta-glucan is useful in adjuvant or supplementation therapy but not as a standard recurrent treatment. Management of the standard cancer treatment protocol is still required [14, 21].

Even though there is no complete evidence that beta-glucan can be effectively used as anti-cancer factors, there are many interesting investigations confirming its usefulness to affect cancer cells in vitro and in vivo [22–24]. Our data showed that the high molecular weight beta-glucan similar to low didn’t affect the normal human keratinocytes in concentration 50 to 200 μg/ml.

In both tested lung cancer cell line we observed the proportional decrease of cell viability after incubation with HMW beta-glucan. The LMW beta-glucan causes only a slight cytotoxicity in these cells. The research of Hong et al. [9] is concentrated on the antitumor effect of glucan from microorganisms (*Paenibacillus polymyxa* JB115) on four different cancer lines (A549, Hela, Hep3B, Sarcoma 180) [9]. Significant cytotoxicity was observed in Hela and Sarcoma 180 cells. The cytotoxicity of beta-glucan was confirmed by Kim et al. [21]. They studied colon cancer cells and postulated that viability of cancer cells is dependent on the applied dose of beta-glucan. They tested with MTT assay usage indicated that 200 μg/ml dose caused decreasing of viability of cancer cells about 50% [21]. Other studies showed in contrast the inhibition rate of beta-(1–3) glucan isolated from *Poria cocos* mycelia on Sarcoma 180 as less than 10% [25, 26]. Moreover Zhang et al. [27] used water-soluble beta-glucan including mainly 1 → 3 and 1 → 4 linkages obtained from the mycelia of *Poria cocos* (PCM3-II). The dose effect of PCM3-II on MCF-7 cell line was studied by incubating these cells with 12.5–400 μg/ml of the glucan for 72 h. In this case the MTT examination showed that PCM3-II reduced proliferation and viability of the MCF-7 cells dose-dependently, so that the cancer-cell growth was reduced by 50% of the control level at 400 μg/ml of the beta-glucan [27].

We found some straight cytotoxic effects of beta-glucan in A549 and H69AR cell line in opposite to other investigation in which any direct decrease of tumor cells proliferation was initiated [6, 28]. We examined the oxidative markers such lipid peroxidation, expression of mitochondrial superoxide dismutase MnSOD and cytoskeletal changes. In contrast to normal human keratinocytes the level of MDA was increased both in human adenocarcinoma lung cell line and in multidrug resistant small cell lung cancer cell line in every concentration. Yamamoto et al. [29] described that beta-glucan from mushroom activated suppression of angiogenesis and metastasis in orally controlled model. Also it is well documented that beta-glucan from mushrooms has reduced pulmonary metastasis and inhibited the growth of metastatic cancer in the lung [29].

Possibly beta-glucan can induces oxidative stress into the tumor cells. The high expression of mitochondrial superoxide dismutase and significant changes in cytoskeleton of A549 and H69AR lung cancer cell line confirm our suggestion. Some research demonstrated that apoptosis is activated in cancer cells by beta-glucan through an increase the expression of caspase-3 enzyme. Additionally beta-glucan can lead to changes morphology and of the expression of proapoptotic gene [21]. The apoptosis death pathways can be activated multifactorial. One of the ways of inducing apoptosis in tumor cells is oxidative stress. Some studies show that bioactive beta-glucan polysaccharide of the Maitake mushroom has cytotoxic outcome probably through oxidative stress on prostatic cancer cells, which lead to apoptosis.

To explore more effective treatment for hormone-refractory prostate cancer, they investigated the potential antitumor effect of beta-glucan, on prostatic cancer cells in vitro. Enhancement of cytotoxic effect of glucan by vitamin C and carmustine can also have clinical application [30]. Previous results show that beta-glucan can induced apoptosis by internal pathway, due to modulation of Bcl-2 family and activation of caspase 3 expression [21]. Soluble beta-glucan from *Candida albicans* induced significantly apoptosis and oxidative stress, enhanced the formation of 8-OHdG and HO-1in the lung isolated from mice, which is associated with lung injury [31]. Kobayashi et al. [22] reported also that beta-glucan from *Agaricus blazei* Murill had cytotoxic effect against human ovarian cancer HRA cells, but not against murine Lewis lung cancer 3LL cells [22].

Bone marrow hemopoietic suppression and decrease of blood cell populations represent major damaging consequences in anticancer chemotherapy. Therefore, we wanted to evaluate the effect of beta-glucan not only on cancer cells but also on normal human red blood cells. Our studies have also showed that the protective effects of beta*-*glucan against hemolysis increased in a dose-dependent manner. The test performed has allowed to demonstrate that relatively low concentrations of beta*-*glucan (300 μg/ml***)*** have been shown to efficiently inhibit the hemolytic action of hypotonic sodium chloride solution and distilled water. It can suggest that hemolysis of the red blood cells can be blocked by virtue of reversible binding of the beta*-*glucan to the erythrocyte membrane. Hemolysis by hypotonic solution of sodium chloride appeared to be inhibited by beta*-*glucan, presumably by a mechanical enhancement mechanism of erythrocyte phospholipid.

The effect of beta*-*glucan is consistent with our suggestion that this plant polysaccharide prevent hemolysis by binding loosely to the erythrocyte, presumably to the erythrocyte surface. Clarification of this issue would require carrying out microscopic examination to show whether the beta-glucan is present in the form of one larger conglomerate or more discrete molecules physically associated with erythrocyte plasma membranes. Possibly in these instances, single beta-glucan molecules appear to encompass the plasma membrane of erythrocytes in the form of network. The morphology analysis of the red blood cells surface would provide confirmation of this thesis that the beta*-*glucan can create insoluble linkages with the red blood cell membrane constituents. It can be seen that HMW beta-glucan was more effective in inhibition of hemolysis. Probably this fraction of beta-glucan has greater avidity for erythrocyte membrane phospholipids.

Presumably the protective effect of the beta*-*glucan in part was dependent also by virtue of impact against lipid peroxidation [32, 33]. Beta*-*glucan is known to have protective effects on antioxidant status and peroxidation of phospholipids. Several studies have shown that oral consumption of beta*-*glucan in human and animals can be effective in preventing oxidative stress which damages other relevant blood components such as platelets [34].

Obtained results allow to suggest that plant polysaccharides as beta-glucan may afford beneficial effects in preventing oxidative damage to membranes of erythrocytes. These observations may be useful in preventing or treating various disease conditions relating to erythrocytes in which lipid peroxidation and mechanical damage to the membrane plays a role. Further studies on beta-glucan are required to find new biochemical and therapeutic properties of it have and assess their effects and mechanisms of actions.

Summing up, based on the test conducted on three cell lines: human lung adenocarcinoma cancer, human multidrug resistant small cell lung cancer and normal human keratinocytes was found that 1–3, 1–4 –D-beta-glucan derived from oats is cytotoxic and induces oxidative stress in cancer cells in comparison to normal. These refers to both investigated the beta-glucan of low and high molecular weight. Our studies have also shown that human erythrocytes treated with beta-glucan (1*–*3*,* 1*–*4*-*beta*-*D*-*glucan) are less susceptible to hemolysis in the hypotonic medium, demonstrating additionally the prospect to use human erythrocytes in various hemolysis experiments. Even though the signaling pathway dependent on beta-glucan is still not fully understood and needs further study, but it is generally known that beta-glucan can be qualified to cancer-preventing and direct tumor inhibition activities.

## Electronic supplementary material

ESM 1 (DOC 58 kb)
