# Supplementary material for: High- and low-Molecular Weight oat Beta-Glucan Reveals Antitumor Activity in Human Epithelial Lung Cancer
Source: Pathol Oncol Res. 2017 Jul 29;24(3):583–92. doi: 10.1007/s12253-017-0278-3 (PMC5972159; doi:10.1007/s12253-017-0278-3)
Supplement: Supplementary file 1 — (DOC 58 kb) [file 12253_2017_278_MOESM1_ESM.doc]

**Supplementary Material**

**Table 1.** Statistical analysis of lipid peroxidation results.

| MDA concentration | ANOVA - source of variation | | | | | | Post hoc | | |
| --- | --- | --- | --- | --- | --- | --- | --- | --- | --- |
| between groups | *SS* | *df* | *MS* | *F* | *P-Value* | *F crit* | LSD | HSD | Scheffe |
| A549 – LMW | 0,011143984 | 9 | 0,00123822 | 137,5800521 | 5,35391 ˄ 10^-16^ | 2,392814108 | 0,005109546 | 0,00867411 | 0,005358523 |
| H69AR – LMW | 0,013917653 | 9 | 0,001546406 | 171,8228748 | 6,04745 ˄ 10^-17^ | 3,956443323 | 0,007724222 | 0,00867411 | 0,006890379 |
| HaCaT – LMW | 0,002017875 | 9 | 0,000224208 | 24,91203399 | 5,66572 ˄ 10^-9^ | 2,392814108 | 0,005109546 | 0,00867411 | 0,005358523 |
| A549 – HMW | 1,081982742 | 9 | 0,120220305 | 42,09885769 | 4,66611 ˄ 10^-11^ | 3,956443323 | 0,137590124 | 0,154510305 | 0,122737027 |
| H69AR – HMW | 1,703209726 | 9 | 0,189245525 | 23,25572782 | 1,04432 ˄ 10^-8^ | 3,956443323 | 0,232263705 | 0,260826392 | 0,207190426 |
| HaCaT - HMW | 1,360664221 | 9 | 0,151184913 | 43,04401066 | 3,79165 ˄10^-11^ | 3,956443323 | 0,152591861 | 0,171356884 | 0,136119299 |

*critical values for the inverse two-sided Student's t-distribution
